# Development of a Murine Infection Model with* Leishmania killicki*, Responsible for Cutaneous Leishmaniosis in Algeria: Application in Pharmacology

**DOI:** 10.1155/2016/7985104

**Published:** 2016-02-02

**Authors:** Naouel Eddaikra, Ihcene Kherachi Djenad, Sihem Benbetka, Razika Benikhlef, Khatima Aït-Oudhia, Farida Moulti-Mati, Bruno Oury, Denis Sereno, Zoubir Harrat

**Affiliations:** ^1^Laboratoire d'Eco-Épidemiologie Parasitaire et Génétique des Populations, Institut Pasteur d'Algerie, Route de Petit Staouéli, Dely Brahim, Algiers, Algeria; ^2^Unité Mixte de Recherche IRD 224 MiVegec (Maladies Infectieuses et Vecteurs: Écologie, Génétique, Évolution et Contrôle), Institut de Recherche pour le Développement (IRD), BP 64501, 34394 Montpellier Cedex 5, France; ^3^Laboratoire de Biochimie Analytique et Biotechnologies, Université Mouloud Mameri de Tizi-Ouzou, Algeria; ^4^Ecole Nationale Supérieure Vétérinaire, Hassan Badi, BP 161, El Harrach, Algiers, Algeria; ^5^Unité Mixte de Recherche IRD 177 InterTryp (“Interactions Hôtes-Vecteurs-Parasites-Environnement dans les Maladies Tropicales Négligées dues aux Trypanosomatides”), Institut de Recherche pour le Développement (IRD), BP 64501, 34394 Montpellier Cedex 5, France

## Abstract

In Algeria,* Leishmania infantum*,* Leishmania major*, and* Leishmania killicki* (*Leishmania tropica*) are responsible for cutaneous leishmaniosis. We established a murine model of* L. killicki* infection to investigate its infective capacity, some immunophysiopathological aspects, and its suitability for pharmacological purposes. Following the injection of* L. major* or* L. killicki* metacyclic promastigotes in the ear dermis of BALB/c mice, the course of infection was followed. The infection with* L. killicki* caused slower lesion formation than with* L. major*. The presence of* L. killicki* or* L. major* DNA and parasites was detected in the ear dermis and in lymph nodes, spleen, and liver. Lesions induced by* L. killicki* were nonulcerative in their aspect, whereas those caused by* L. major* were highly ulcerative and necrotic, which matches well with the lesion phenotype reported in humans for* L. killicki* and* L. major*, respectively. The treatment of* L. killicki* lesions by injection of Glucantime® significantly reduced the lesion thickness and parasite burden. Ear dermal injection of BALB/c mice constitutes a model to study lesions physiopathology caused by* L. killicki* and presents interest for* in vivo* screening of new compounds against this pathogen, emerging in Algeria.

## 1. Introduction

*Leishmania* are obligate intracellular parasites, which cause different forms of leishmanioses in humans, ranging from dermal ulcers to fatal visceral forms. Cutaneous leishmanioses (CL) are caused by several* Leishmania* species and display various clinical manifestations. In Algeria,* L. killicki* was discovered in 2005 in the southern province of Ghardaia and recently reported in the northern part of the country [1, 2]. Phylogenetic studies based on Multilocus Enzyme Electrophoresis showed that* L. killicki* strains were included in clearly distinct clades within the* L. tropica* complex. A recent Multilocus Sequence Analysis further evidenced the substructuration of* L. tropica* species and supported this proposal [3, 4]. In Algeria, up till now only strains belonging to the* L. killicki* subgroup within the* L. tropica *complex have been isolated. The pathogenicity and the infectivity of these strains have never been studied. Cutaneous lesions caused by* L. tropica* tend to form dry ulcers that require a long time to heal, typically one year or more. Healing is often associated with disfiguring scars and papules that can also recur at the periphery of the original lesion and are called recurrent type lesions [5, 6]. Cutaneous lesions caused by* L. killicki* are called chronic cutaneous lesions because they persist for several years [7]. So clinical signs of cutaneous leishmaniosis due to* L. killicki *are restricted to a chronic cutaneous lesion, resistant to standard treatment in opposite to* L. tropica *cutaneous leishmaniosis [8–11]. In addition, the transmission of* L. killicki* seems to be strictly zoonotic, while it is anthroponotic or occasionally anthropozoonotic for* L. tropica*.

Animal models have been used in the drug discovery and development process to characterize disease physiopathology and to estimate clinical dosing regimens safety margins and toxicity and of course to validate targets and compounds. The ideal in an animal model is that it should replicate to a great extent a human disease phenotype and its underlying causality. Many experimental models have been developed on rodents to study CL. Mouse models were established for* L. major*,* L. tropica*,* L. amazonensis*, and* L. braziliensis*, each with specific features in order to characterize the immune response, but none of them reproduces the pathology observed in human disease [12–14]. For* L. tropica* and* L. major*, patterns of responses are species specific with different sex effects and largely different host susceptibility genes [6, 15, 16]. So,* L. killicki* causes in humans cutaneous lesions, which tend to form dry ulcers similar in their aspect to those caused by* L. tropica* [1, 6, 7]. Nevertheless, their healing requires a longer period of treatment. CL caused by* L. killicki* is called chronic CL because lesions persist for years, as opposed to CL caused by* L. major* for which lesions usually resolve with scare after few months only [7].

The aims of this work were therefore to develop an animal model that allows us to study basic physiopathological and immunological aspects of the infection caused by* L. killicki* and to evaluate its suitability for pharmacological purposes.

## 2. Materials and Methods

### 2.1. Parasites

*L. major* (MHOM/DZ/10/LIPA175/11) and* L. killicki* (MHOM/DZ/11/LIPA281/11) were maintained in RPMI 1640 medium supplemented with 10% heat-inactivated FBS, 2 mM L-glutamine, 25 mM glucose, 100 *μ*g/mL streptomycin, and 100 IU/mL penicillin at 25°C. Strain virulence was maintained by a regular passage in susceptible BALB/c mice.

### 2.2. Ethics Statement

All experiments were carried out in compliance with the guidelines of the Federation of European Laboratory Animal Science Associations (FELASA) and approved by the Ethical Committee of the Pasteur Institute in Algiers.

### 2.3. Mouse Infection

A total of 120 BALB/c mice were obtained from the animal breeding stock facility of Pasteur Institute in Algiers. Six-week-old females were kept in conventional conditions with barriers, controlled temperature, and light cycle. Food and water were provided* ad libitum*. Infective promastigotes were isolated at the metacyclic stage from stationary phase cultures (6 days old). Metacyclic promastigotes were isolated on a Ficoll gradient [17], washed once with phosphate buffered saline (PBS) (pH 7.3), and then resuspended in PBS (pH 7.3). 10^3^ metacyclic promastigotes in 10 *μ*L of PBS were injected in the left ear of each mouse [13].

### 2.4. *L. killicki* Animal Model

To establish an* in vivo* model of cutaneous lesions caused by* L. killicki*, we compared the appearance and the induration thickness of the lesion following intradermal inoculation with* L. killicki* or* L. major* into the ear dermis of BALB/c mice. To monitor lesion development, mice were divided into three groups: 5 mice for the control noninfected mice, 5 mice infected with* L. major*, and 5 mice infected with* L. killicki*. Experiments were conducted during 12 consecutive weeks after infection in mice infected with* L. major* and during 30 consecutive weeks after infection for mice infected with* L. killicki*. Lesion development was monitored by measuring the ear thickness using a digital micrometer caliper (Fisherbrand) at weeks 2, 4, 6, 10, and 12 after infection for* L. major* and at weeks 2, 4, 6, 8, 10, 12, 14, 16, 18, 20, and 30 after infection for* L. killicki*. Results are expressed as the difference between thicknesses of the inoculated ear and the noninoculated contralateral ear (internal control).

### 2.5. Detection and Quantification of Parasites in Tissues

To look at the capacity of* L. killicki* and of* L. major* to disseminate into internal organ, the presence of parasite DNA and of live parasites was investigated at weeks 2, 4, 8, and 12 for* L. major* infected mice and at weeks 2, 4, 8, 12, 16, and 20 for the* L. killicki* group. Mice were euthanized, and the retromaxillary draining lymph nodes, spleen, and liver were collected to extract DNA. At the 12th week, an aliquot from each sample was seeded to LIT (Liver Infusion Tryptose) medium and incubated for 4 weeks in order to isolate live parasites. Samples of tissue were homogenized in PBS using potter grinders and 1.5 mL microtubes with single-use blue pellet pestles (Polylabo, France). Homogenates were then aliquoted and stored at −20°C until DNA extraction. Total DNA was purified using the QIAamp DNA mini kit (QIAGEN) according to the manufacturer's protocol. Parasite DNA was detected after amplification of the ribosomal internal transcribed spacer 1 (ITS1) using primers LITSR and L5.8S previously designed by Schönian et al. [18]. The PCR mix (25 *μ*L) contained 2.5 *μ*L of DNA, 10x buffer, 300 *μ*M MgCl_2_, 200 *μ*M dNTP, 500 nM of each primer, and 2 U of Taq DNA polymerase. Amplification products were separated on a 1% agarose gel and visualized after staining with ethidium bromide.

To investigate parasite proliferation during lesion expansion, we measured the parasite load at the inoculation site. At weeks 4, 8, and 12 for* L. major* infected mice and at weeks 4, 8, 12, 16, 20, and 30 for* L. killicki* groups, mice were euthanized. Parasite proliferation within the lesion was monitored by counting the number of amastigotes in Giemsa-stained smears under 100x magnification. The number of infected macrophages and the mean number of amastigotes per macrophage were determined in one hundred randomly selected fields. The results are expressed as the mean number of parasites per 100 macrophages.

### 2.6. Drug Treatment

In a first attempt to investigate the capacity of this new* in vivo* murine model of infection with* L. killicki* to be used for the screening of new antileishmanial compounds, we compared the outcome of Glucantime treatment which consisted in injecting drug into lesions at weeks 4 and 8 for* L. major* and* L. killicki*, respectively. In all experiments, the treatment was initiated when the infection was well established and when the lesions were obvious 4 and 8 weeks after the inoculation with* L. major* and* L. killicki*, respectively. Two days before drug administration, mice were randomly divided into 2 groups of thirty animals. N-Methylglucamine antimoniate (pentavalent antimony: SbV) was diluted in PBS and then administered to mice by injection directly into lesions at a dose of 28 mg per kg of body weight every 5 days for 15 days. Ear thickness was measured weekly during the treatment and after the end of the treatment. The antimony treatment efficiency was monitored by calculating ear thickness and parasite load indexes:

 Ear thickness index = mean thickness of ear from treated mice/mean thickness of ear from untreated mice.
 Parasite load index = mean parasite load in untreated mice/mean parasite load in treated mice.

### 2.7. Statistical Analysis

Values are given as the mean ± SEM for groups of *n* samples. Analysis of variance (ANOVA) and Student's *t*-test were performed using GraphPad Prism Software (GraphPad Software Inc., San Diego, California, USA) and Microsoft Office Excel 2013 was used to determine the significance of differences.

## 3. Results

### 3.1. Lesion Appearance and Development

A striking difference in the onset, the type, and the severity of lesions was observed between both* Leishmania *species. Cutaneous redness, which is the first symptom of infection caused by tissue inflammation, was detectable 2 weeks after the infection of mice with* L. major* but 4 weeks after infection with* L. killicki*.

**Figure 1:**
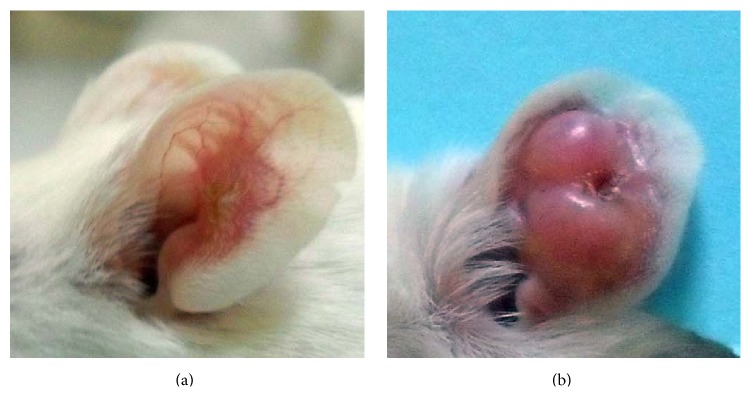
Clinical evolution of the ear lesions in BALB/c mice. Lesions' appearance in mice infected with* L. major *(4th week after infection) (a) or* L. killicki* (20th week after infection) (b).

After 4 weeks,* L. major*-infected mice exhibited lesions with elevated borders and sharp craters (Figure 1(a)). Mice infected with* L. killicki* developed a detectable lesion later: the ear thickness increased progressively during the time course of the experiment (Figure 1(b)). Infection never caused lesion ulceration, which was observed in* L. major*-infected mice. Strikingly, lesion phenotypes induced by* L. killicki* were clearly distinct from those induced by* L. major*.

**Figure 2:**
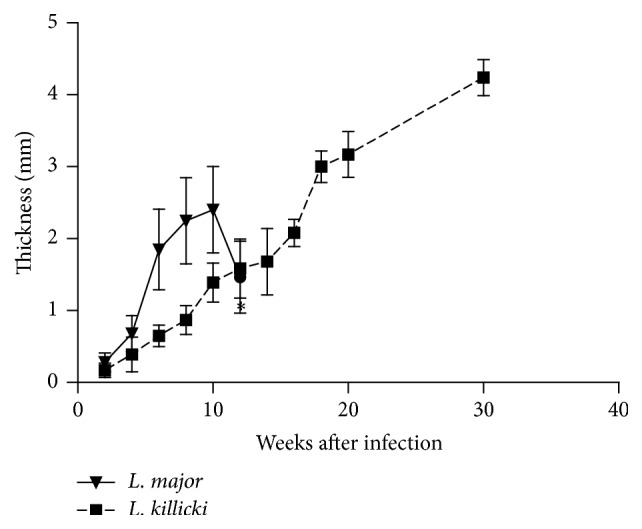
Evolution of indurations following infection of BALB/c mice with* Leishmania killicki* or* Leishmania major.* The ear thickness is expressed as the difference between the thicknesses of the inoculated ear and the noninoculated contralateral ear. The data represent the mean values of measures ± standard deviations (*n* = 5). Note: 12 weeks (*∗*) after infection with* L. major*, ears became necrotic and showed a loss of tissue which has prompted us to interrupt the experiment at this time.

In mice infected by* L. major* metacyclic promastigotes, thickening of the ear was observed at the inoculation site as early as 3 weeks after infection. The ear thickness rapidly increased, reached a maximum of 2.4 mm at week 10, and then regressed (Figure 2). For* L. killicki*, the ear thickness expanded linearly and more slowly throughout the time course of the experiment to reach 1.4 mm and 4.3 mm at weeks 10 and 30, respectively.

### 3.2. Parasite Burden in the Ear Dermis and Occurrence in Other Organs or Tissues

**Figure 3:**
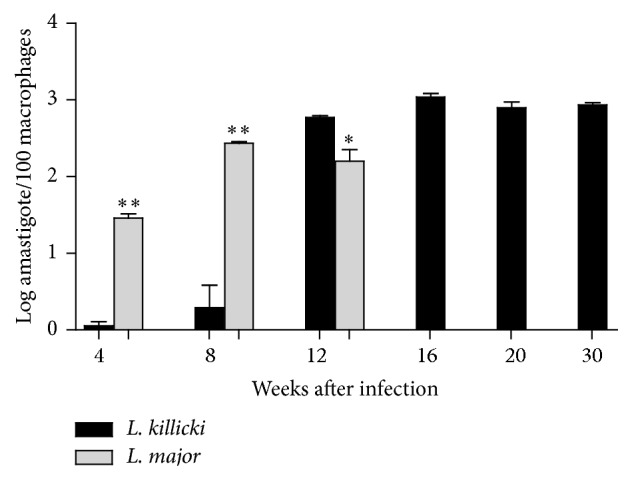
Parasite burden measured in ears of BALB/c mice infected with* Leishmania killicki* or* Leishmania major*. Following intradermal injection of 10^3^ metacyclic promastigotes, the parasite load was estimated as described in Materials and Methods. Each bar is representative of the mean of five determinations ± standard deviations. Statistical analysis (^*∗*^ *p* < 0.05 and ^*∗∗*^ *p* < 0.01) was performed using Student's *t*-test under GraphPad Prism (*n* = 5 mice/group).

The presence of parasites in the ear dermis of mice inoculated with* L. major* was observed as early as the 4th week (Figure 3). Moreover, the parasite burden steadily increased until the 8th week and the maximum was reached at the 10th week (Figures 1 and 3). As from this time, the ear thickness and the lesion size slightly decreased until the ear perforated.

The onset of parasite burden in* L. killicki*-infected mice was different. Parasite expansion began later than for* L. major*, 8 weeks after infection, reached its maximum at week 16, and was maintained until the end of the experiment at week 30. The maximal parasite burden was significantly higher than for* L. major*-infected mice (*p* < 0.001). In* L. killicki*-infected mice, no further parasite expansion was observed after the 12th week, while the induration thickness increased (Figures 1 and 3).

Beyond the capacity of* Leishmania* to replicate at the inoculation site, the dissemination of* L. killicki* and* L. major* in various tissues or organs was further investigated (Table 1).* L. major* and* L. killicki* were both detected in culture at 12 weeks. Indeed, DNA detection evidenced the presence of parasites at the inoculation site from the 2nd week after infection for both species. However, the major difference was the delay observed for the colonization of the organ following infection.* L. major* colonized draining lymph nodes and spleen more quickly, 2 weeks after infection compared to 4 weeks for* L. killicki* (Table 1). DNA was detected in liver 4 weeks after infection for both species. After this time point, the PCR remained positive until the 12th week for* L. major* and the 20th week for* L. killicki*.

### 3.3. Compared Clinical Evolution of* L. killicki* and* L. major* Lesions and Parasite Load under Antimonial Treatment

**Figure 4:**
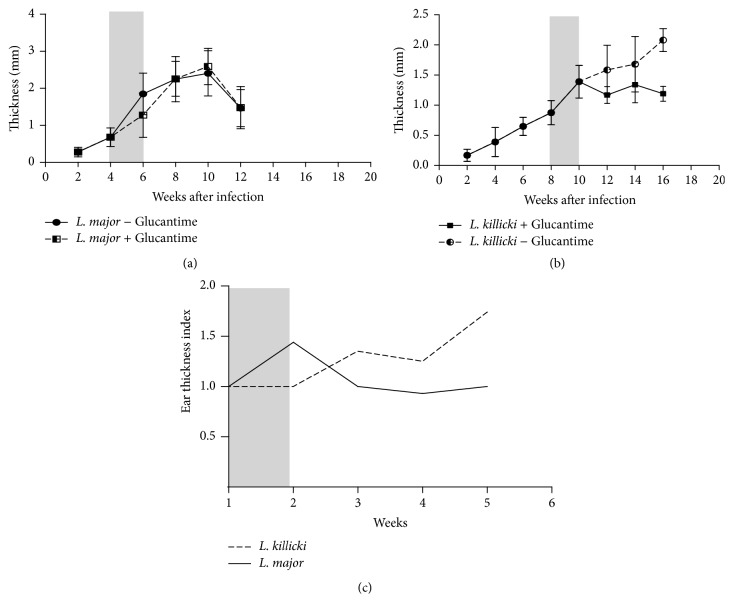
Effect of Glucantime treatment on ear induration in mice infected with* Leishmania major* (a) or* Leishmania killicki* (b) and on the ear thickness index (c). Grey bars indicate the Glucantime treatment period. The induration thickness is expressed as the difference of thicknesses between infected ears and contralateral noninoculated ear (control). Data are expressed as mean values ± standard deviations (error bars) (*n* = 5).

The ear thickness was roughly similar in* L. major*-infected mice when treated with drug or PBS (Figure 4(a)). The reduction in the lesions observed in* L. major*-infected mice 10 weeks after infection was due to tissue necrosis and loss. These results show that antimony has no or undetectable effect on* L. major* lesions after the end of the treatment, although at concentration we used in our experiment (Figure 4(c)). Interestingly, the expansion of lesion halted in treated* L. killicki*-infected mice submitted to chemotherapy (Figure 4(b)). Accordingly, the ear thickness index increased constantly during the time course of the treatment (Figure 4(c)).

**Figure 5:**
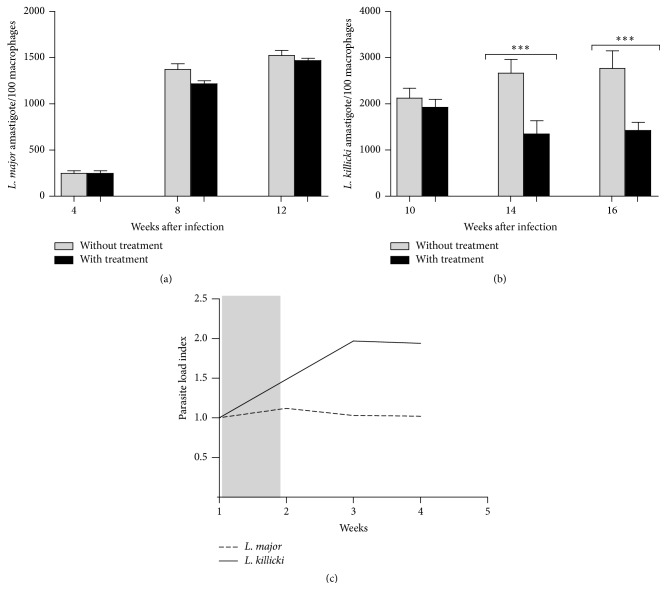
Parasite load in mice infected with* Leishmania major* (a) or* Leishmania killicki* (b) and evolution of the parasite load index following antimony treatment (c). The parasite load index was calculated as follows: mean parasite load in untreated mice/mean parasite load in treated mice. Each bar is representative of the mean parasite load determined in 5 mice ± standard deviations (error bars). Statistical analysis (^*∗∗∗*^ *p* < 0.01) was performed using Student's *t*-test under GraphPad Prism (*n* = 5 mice/group). Grey bar indicates the Glucantime treatment period.

Glucantime treatment slightly affects the parasite load with only 0.12-fold reduction of the mean number of* L. major* amastigotes/100 macrophages in lesions (see Figures 5(a) and 5(c)). However, it is more efficient in* L. killicki*-infected mice, which exhibited twofold reduction in* L. killicki* amastigotes (Figures 5(b) and 5(c)). Parasite loads were significantly different as early as one week after the beginning of the treatment, that is, at the third injection of Glucantime (*p* < 0.001). Overall, a better concordance between parasite load and ear thickness was observed in mice infected with* L. killicki* during antimony treatment than in mice infected with* L. major*.

## 4. Discussion

In this work we seek for the first time to establish a CL animal model for the emerging* Leishmania* parasite in Algeria:* L. killicki*. In an attempt to reproduce the natural biology of* Leishmania* transmission, Belkaid et al. [19] established a dermal model of infection in which low numbers of* L. major* promastigotes were injected into the ear. Based on this methodology, we established a dermal model of infection using* L. killicki,* which involved a cutaneous lesion in the ear dermis of mice, similar to those observed in patients with* L. killicki* infection, that is, localized lesions that do not heal spontaneously. The appearance of lesions induced by* L. killicki* is different from those produced by* L. major* or* L. tropica sensu stricto* [6, 16]. Experimental infection of BALB/c mice with* L. tropica* produced lesions that developed up to 3 months after infection and then regressed [6, 16]. In mice infected with* L. killicki*, we never observed regression of the ear thickness but a continuous extension of the lesion. Indeed, in humans,* L. killicki* induces chronic lesions that persist up to one year [2]. In the mouse, we observed that* L. killicki* lesions do not spontaneously heal but persist and develop all along the experiment. Further studies will be required to understand the underlying immunological determinants allowing the long-term persistence of* L. killicki* in lesions. In the old world, only* L. infantum* and* L. donovani* are known to cause visceral forms of leishmaniosis (VL). Nevertheless, these two species can also be the causative agents of some forms of CL [20, 21]. In the same way,* L. tropica* as* L. major* cause CL in humans but different studies reported the isolation and characterization of* L. tropica* in patients with VL [8, 22–24]. In mice,* L. tropica* or* L. major* are also known to cause visceral infections; that is, they are detected in spleen and liver but the rapidity of the visceral dissemination, the symptoms observed, and the parasite load differ between* L. tropica* and* L. major* [25–27]. We observed that* L. killicki* had also the capacity to disseminate and to persist in internal organs of mice. Nevertheless, the ability of* L. killicki* to cause VL in humans remains unknown and has not been reported to date.

*L. killicki* is considered as an emerging pathogen resistant to pentavalent antimonial treatment [1, 2, 7, 10]. Therefore, it would be interesting to evaluate the suitability of this* L. killicki* infection model in experimental pharmacology. This infection model could test the leishmanicidal activity of known drugs and be predictive of their clinical efficacy. Currently, animal models of infection optimized to test antileishmanial compounds are available for* L. major* and* L. amazonensis* [28, 29]. Human infections caused by* L. tropica *complex are considered to be refractory to most classical treatments, including antimonial containing drugs, unlike* L. major* infections [22, 30, 31]. In our study, we have observed that antimony treatment does not affect the outcome of lesions induced by* L. major* in contrast to* L. killicki*. In fact, the outcome of the experiment cannot be further followed in mice infected with* L. major *because of lesions' necrosis. The treatment of mice infected with* L. killicki* resulted in the reduction of lesions size and of the thickness which were associated with a drastic diminution of the parasite load at the inoculation site.

## 5. Conclusions

Our observations support the notion that this* L. killicki* model of infection has several practical advantages over the* L. major* model. First, the drug regimen can be evaluated over a longer time period (up to 30 weeks) as compared to* L. major*, where tissue loss and the appearance of ulcerative lesions limit the time course of the experimentation. Second, the intense and continuous parasite multiplication at the inoculation site makes it possible to more easily assess the leishmanicidal activity of new molecules in a simple way. The continuous emergence of antimony resistance in* Leishmania* spp. in various parts of the world necessitates the development of new alternative antileishmanial drugs [32–34]. To this end, this study supports the notion that this new* L. killicki* experimental model might be useful for screening and validating new compounds* in vivo*.

## Figures and Tables

**Table 1:** Detection of *L*. *major* and *L*. *killicki* DNA and parasitesin various tissues of BALB/c mice. ND, not determined.

*Leishmania*	Tissue or organ	PCR after inoculation/LIT culture					
		2 weeks	4 weeks	8 weeks	12 weeks	16 weeks	20 weeks
*L. major*	Ear (inoculation site)	+	+	+	+/ND		
	Draining lymph node	+	+	+	+/+		
	Spleen	+	+	+	+/+		
	Liver	−	+	+	+/+		

*L. killicki*	Ear (inoculation site)	+	+	+	+/ND	+	+
	Draining lymph node	−	+	+	+/+	+	+
	Spleen	−	+	+	+/+	+	+
	Liver	−	+	+	+/+	ND	ND
